# Sex differences in the influence of type 2 diabetes (T2D)-related genes, parental history of T2D, and obesity on T2D development: a case–control study

**DOI:** 10.1186/s13293-023-00521-y

**Published:** 2023-06-08

**Authors:** Jaime Berumen, Lorena Orozco, Héctor Gallardo-Rincón, Fernando Rivas, Elizabeth Barrera, Rosa E. Benuto, Humberto García-Ortiz, Melissa Marin-Medina, Eligia Juárez-Torres, Anabel Alvarado-Silva, Espiridión Ramos-Martinez, Luis Alberto MartÍnez-Juárez, Julieta Lomelín-Gascón, Alejandra Montoya, Janinne Ortega-Montiel, Diego-Abelardo Alvarez-Hernández, Jorge Larriva-Shad, Roberto Tapia-Conyer

**Affiliations:** 1 Unidad de Investigación en Medicina Experimental, Facultad de Medicina, Universidad Nacional Autónoma de México, Cuauhtémoc, 06720 Ciudad de Mexico, México; 2 Instituto Nacional de Medicina Genómica, Ciudad de Mexico, México; 3 Universidad of Guadalajara, Health Sciences University Center, Guadalajara, Jalisco México; 4 Fundación Carlos Slim, Lago Zurich 245, Presa Falcon Building (Floor 20), Col. Ampliacion Granada, Miguel Hidalgo, 11529 Mexico City, México; 5 Laboratorio Huella Génica, Ciudad de Mexico, México; 6 Instituto de Neurobiología, Universidad Nacional Autónoma de México, Juriquilla, Querétaro México; 7 Facultad de Medicina, Universidad Nacional Autónoma de México, Ciudad de Mexico, México

**Keywords:** Age, Genetic loci, Obesity, Sex, Type 2 diabetes, Parental history

## Abstract

**Background:**

This study investigated the effect of sex and age at type 2 diabetes (T2D) diagnosis on the influence of T2D-related genes, parental history of T2D, and obesity on T2D development.

**Methods:**

In this case–control study, 1012 T2D cases and 1008 healthy subjects were selected from the Diabetes in Mexico Study database. Participants were stratified by sex and age at T2D diagnosis (early, ≤ 45 years; late, ≥ 46 years). Sixty-nine T2D-associated single nucleotide polymorphisms were explored and the percentage contribution (*R*^2^) of T2D-related genes, parental history of T2D, and obesity (body mass index [BMI] and waist–hip ratio [WHR]) on T2D development was calculated using univariate and multivariate logistic regression models.

**Results:**

T2D-related genes influenced T2D development most in males who were diagnosed early (*R*^2^ = 23.5%; females, *R*^2^ = 13.5%; males and females diagnosed late, *R*^2^ = 11.9% and *R*^2^ = 7.3%, respectively). With an early diagnosis, insulin production-related genes were more influential in males (76.0% of *R*^2^) while peripheral insulin resistance-associated genes were more influential in females (52.3% of *R*^2^). With a late diagnosis, insulin production-related genes from chromosome region 11p15.5 notably influenced males while peripheral insulin resistance and genes associated with inflammation and other processes notably influenced females. Influence of parental history was higher among those diagnosed early (males, 19.9%; females, 17.5%) versus late (males, 6.4%; females, 5,3%). Unilateral maternal T2D history was more influential than paternal T2D history. BMI influenced T2D development for all, while WHR exclusively influenced males.

**Conclusions:**

The influence of T2D-related genes, maternal T2D history, and fat distribution on T2D development was greater in males than females.

**Supplementary Information:**

The online version contains supplementary material available at 10.1186/s13293-023-00521-y.

## Plain Language summary

The prevalence of diabetes worldwide is slightly higher in men than in women, particularly in those aged 50 or younger (16.5% for men versus 13.5% for women). This suggests that hormonal differences could be critical in early development of Type 2 diabetes. Some known factors previously associated with T2D, such as genes, parental history of diabetes and obesity, could have a differential influence between both sexes for the development of T2D. We compared these factors between 1008 healthy individual and 1012 TD2 patients. In this comparison, we calculated the percentage of variability of the disease explained by each factor. As expected, the most noticeable differences between men and women were observed in T2D diagnoses before age 46. Genes had a greater effect in men than in women (23.5% vs. 13.5%). While genes involved in insulin production have a greater influence on men, genes involved in peripheric insulin resistance have a greater influence on women. The overall parental history of T2D influences similarly in males (19.9%) and females (17.5%), however, the unilateral genetic influence of the mother was much greater in males than in females. The influence of global and abdominal obesity played a greater role in men than in women. In T2D diagnoses after age of 45, the influence of genes and parental history of diabetes decreases markedly, and the relative influence of global obesity augments. However, while genes linked to insulin resistance and inflammation predominate in females, genes linked to insulin secretion predominate in males.

**Supplementary Information:**

The online version contains supplementary material available at 10.1186/s13293-023-00521-y.

## Highlights

- The influence of T2D-related genes, maternal T2D history, and fat distribution on T2D development, was greater in males than in females of Latin American mestizo origin.
- Influence of genes and parental history was highest among those with an early diagnosis. In those diagnosed late, the influence of genes and family history of T2D decreased drastically, while the influence of obesity increased.
- With an early diagnosis, insulin production-related genes (from chromosome region 11p15.5 and other genomic regions) were more influential in males while peripheral insulin resistance-associated genes were more influential in females.
- With a late diagnosis, insulin production-related genes from chromosome region 11p15.5 notably influenced males while peripheral insulin resistance and genes associated with inflammation and other processes notably influenced females.
- In males, there was a linear correlation between BMI and WHR and the number of risk alleles in several genes associated with insulin production.

**Supplementary Information:**

The online version contains supplementary material available at 10.1186/s13293-023-00521-y.

## Background

Type 2 diabetes (T2D) is associated with obesity, parental history of diabetes, and genes, among other factors [1]. Obesity, a modifiable factor, is considered the main risk factor, given that T2D risk increases linearly with increased body mass index (BMI) [2–4]. However, the relationship between T2D and obesity has not yet been fully elucidated. Although two-thirds of people with T2D are overweight or obese, only 2–13% of obese people develop T2D. A recent study reported a variability (*R*^2^) of 12.6–14.9% of T2D attributable to obesity using univariate logistic regression (ULR) models. However, these values vary substantially when multivariate logistic regression (MLR) models include parental history of T2D and genes as variables [5].

The importance of parental history on T2D development is well established [6–9], with the heritability of T2D varying between 20 and 25% [6, 7, 9–12]. There are over 400 genes with an identified association with T2D [13]; however, most have very little influence on T2D development. Most studies suggest that gene involvement contributes to no more than 12% of T2D variability [5, 13, 14]. Nevertheless, parental history and genes seem to have a greater influence on T2D when it occurs before the age of 46 years, and this influence decreases substantially when T2D is diagnosed later in life (≥ 46 years) [5, 15]. A previous study in the Mexican population [5] also found an important difference in the association of genes, but not parental history, with T2D between males (*R*^2^ = 11.2%) and females (*R*^2^ = 4.1%). Given that the differences in gene association between sexes have not been previously reported or only reported for individual genes [16, 17], the differences are likely to be specific to the ancestry of populations in Mexico. For instance, polymorphisms (located in *SLC16A11* [18], *INS-IGF2* [19], and *HNF1A* [20]) strongly associated with T2D have been discovered in Mexican and Latin populations that are non-existent/very rare in European populations. Polymorphisms in well-established genes associated with T2D in multiple populations, such as *KCNQ1* and *TCF7L2*, are also important contributors to the difference between sexes [5].

There is little information investigating which biological processes associated with T2D (e.g., insulin production, peripheral insulin resistance, inflammation) are most relevant for the genetic differences between the sexes and the time of T2D onset. This case–control study was conducted to assess the influence of genes on T2D between the sexes, identify the different biological processes and their weights of contribution to T2D between sexes and age at T2D presentation, and understand the change in the contribution of each biological process in each group when obesity and parental history of diabetes are included in MLR models.

## Methods

### Sample selection and study design

The individuals included in this case–control study were part of the Diabetes in Mexico Study (DMS) [21], the study design of which was previously described as part of the SIGMA Type 2 Diabetes Consortium [20]. Briefly, participants were recruited from two tertiary-level hospitals in Mexico City, and T2D was diagnosed according to the American Diabetes Association criteria [22]. This study included 1012 cases (unrelated individuals, > 20 years old, with a previous diagnosis of T2D or fasting glucose levels > 125 mg/dL) and 1008 controls (healthy subjects, > 50 years old, fasting glucose levels < 100 mg/dL) from the DMS database. The cases were sex and hospital-matched with controls, recruited between November 2009 and August 2013. Clinical information collected included weight, waist and hip circumference, and parental history of T2D. For fasting glucose measurements and DNA extraction, 10 mL of intravenous blood was collected. Regression models were used to assess the association of 69 selected single nucleotide polymorphisms (SNPs), parental history of T2D, BMI, and waist-to-hip ratio (WHR) with T2D.

### SNP selection and genotyping

Sixty-nine SNPs associated with T2D were selected, nine associated with T2D in a previous study [5] and 60 from different GWAS databases and published articles (Additional file 1: Table S1). SNPs with a minor allele frequency (MAF) ≥ 10% and odds ratios (OR) ≥ 1.2 or ≤ 0.83 in the Latin American mestizo population and those localized in genes with a role in cellular processes involved in T2D development were prioritized. The SNPs were classified into four groups according to the function and location of the gene in which they occur: Group 1, insulin production located in chromosome region 11p15.5; Group 2, insulin production located in other regions; Group 3, peripheral insulin resistance; and Group 4, inflammation and other functions (Table 1; Additional file 1: Table S2).

**Table 1:** Participant demographic and clinical characteristics (*N* = 2020)

Variable	Females		Males		Both sexes	
	(*n* = 1089)		(*n* = 931)		(*n* = 2020)	
	Control	Cases	Control	Cases	Control	Cases
	(*n* = 543)	(*n* = 546)	(*n* = 465)	(*n* = 466)	(*n* = 1008)	(*n* = 1012)
Continuous variables: means ± SD (*n*)						
Age (years)	59.7 ± 11.1 (543)	55.2 ± 12 (546)^e^	58.6 ± 11.4 (465)	55.7 ± 11.4 (466)^e^	59.1 ± 11.3 (1008)	55.5 ± 11.7 (1012)^e^
BMI_adj_ (kg/m^2^)	28 ± 5 (543)	31.4 ± 5.6 (546)^e^	26.9 ± 4.1 (465)	30.5 ± 5 (466)^e^	27.5 ± 4.6 (1008)	31 ± 5.3 (1012)^e^
Waist (cm)	93.4 ± 11.4 (357)	97.5 ± 11.5 (426)^e^	93.4 ± 10.5 (310)	98.6 ± 12.6 (281)^e^	93.4 ± 11 (667)	97.9 ± 12 (707)^e^
Hip (cm)	103.8 ± 11.2 (336)	106.5 ± 11.9 (424)^d^	98.9 ± 7.9 (298)	101.7 ± 10.8 (277)^d^	101.5 ± 10.1 (634)	104.6 ± 11.7 (701)^e^
WHR	0.9 ± 0.1 (336)	0.92 ± 0.1 (424)^d^	0.94 ± 0.1 (298)	0.97 ± 0.1 (277)^e^	0.92 ± 0.1 (634)	0.94 ± 0.1 (701)^e^
Age at diabetes diagnosis (years)		45.6 ± 10.5 (546)		46.1 ± 10.9 (466)		45.8 ± 10.7 (1012)
Years with the disease		9.5 ± 8.5 (546)		9.6 ± 9 (466)		9.5 ± 8.7 (1012)
Parental diabetes history: % (*n*)						
None	63.5 (273)	37.6 (180)^e^	71.4 (260)	41.7 (174)^e^	67.1 (533)	39.5 (354)^e^
Mother	19.5 (84)	30.1 (144)	14.6 (53)	26.4 (110)	17.3 (137)	28.3 (254)
Father	11.4 (49)	13.6 (65)	9.6 (35)	14.9 (62)	10.6 (84)	14.2 (127)
Both parents	5.6 (24)	18.8 (90)	4.4 (16)	17 (71)	5 (40)	18 (161)
Total	100 (430)	100 (479)	100 (364)	100 (417)	100 (794)	100 (896)
Smoking: % (*n*)						
No	87.9 (515)	88.5 (45)^a^	71.4 (445)	69.5 (57)^a^	80.2 (960)	79.8 (102)^a^
Yes	12.1 (1)	11.5 (479)	28.6 (2)	30.5 (388)	19.8 (3)	20.2 (867)
Total	100 (519)	100 (541)	100 (448)	100 (462)	100 (967)	100 (1003)
T2D treatment: % (*n*)						
No		8.6 (45)		12.8 (57)		10.5 (102)
Yes		91.4 (479)		87.2 (388)		89.5 (867)
Total		100 (524)		100 (445)		100 (969)

*BMI* body mass index, *SD* standard deviation, *T2D* type 2 diabetes, *WHR* waist–hip ratio

BMI_adj_ shows the BMI adjusted as described previously [5]; waist and hip circumferences shown are those measured at enrollment

For the differences of means between groups, the *P* values were assessed using a *t*-test

For the differences in the frequency distribution between groups, the *P* values were assessed using the Chi-square test, using 2 × *N* tables

^a^*P* > 0.05; ^b^*P* < 0.05; ^c^*P* < 0.01; ^d^*P* < 0.001; ^e^*P* < 0.0001

All DNA samples were genotyped for the 69 SNPs using Applied Biosystems TaqMan SNP assay design technology (Foster City, CA, USA). Genotyping was performed by the allelic discrimination assay-by-design TaqMan^®^ method on OpenArray^®^ plates. The plates were analyzed on the QuantStudio™ 12K Flex Real-Time PCR System (ThermoFisher Scientific, Waltham, MA, USA). The genotypes were analyzed using the Genotyper™ Software v1.3 (ThermoFisher Scientific, Waltham, MA, USA).

### Statistical analyses

When determining the sample size, we considered MLR models to have good performance when there was a baseline of 50 cases (as in the ULR models) and 10–15 additional cases for each variable introduced in the model [23]. Because we planned to introduce 15–20 variables in the MLR models, we calculated a minimum number of 200–350 cases in the comparison groups.

Participant age, age at T2D diagnosis, BMI, and WHR were expressed as mean ± standard deviation (SD). The BMI was adjusted (BMI_adj_) for participants who had been diagnosed with T2D for ≥ 3 years using data from patients who had been diagnosed with T2D for ≤ 2 years [5]. The distribution of the frequency of genotypes was assessed according to the Hardy–Weinberg law, based on the allelic frequency and the formula: (*a* + *b*)^2^ = *a*^2^ + 2ab + *b*^2^, where *a* and *b* are the allelic frequencies in the control group. Differences in the distribution of genotypes between the observed and expected results were calculated using the Chi-square test.

To identify the factors associated with T2D in each group, ULR models were used. Variables with *P* < 0.20 in the ULR analysis were considered for entry in the MLR model. From the 69 SNPs explored only 38 passed to the second step (MLR). Finally, 23 of them remained in MLR models. In the ULR and MLR models, case (diagnosis of T2D) or control was considered a dependent variable; values of the alleles and genotypes of the SNPs, parental history, BMI, and WHR were considered explanatory variables. Interactions between each SNP and sex were assessed in the ULR models. All variables (BMI_adj_, WHR, parental history of T2D, and SNPs) were analyzed with stratification by sex (male and female) and age at T2D diagnosis (≤ 45 years and ≥ 46 years) (median age at T2D diagnosis = 45 years). The risk conferred by each factor was calculated by comparing cases and controls using ULR models. Association was expressed as an OR with 95% confidence intervals (CIs); the contribution to the variability of T2D was expressed as Nagelkerke’s *R*^2^, representing the percentage of T2D variability explained by a named factor [24].

Confounders were identified using a theoretical strategy based on a backstep, stepwise MLR model and the change-in-estimate criterion. Confounders were defined as those variables for which the percentage difference between the values of the regression β between the adjusted and non-adjusted variables in the stepwise MLR model was larger than 10% (*P* > 0.1). Therefore, the total variability and the contribution of each factor on T2D was calculated using this MLR model. Genes (grouped by biological processes) and the remaining factors were included successively in the model in different blocks, and the contribution of each factor to the model was assessed by the increase of R^2^ and the decrease in the − 2 log likelihood ratio value from one block to the next; the Omnibus test was used to determine whether the differences between the successive blocks were statistically significant. The variation in the order of entry of each factor allowed us to identify how obesity and parental history affect the importance of gene biological processes linked to insulin production and resistance in the variability of T2D between sexes and age at disease onset. A post hoc power analysis was performed for each logistic regression model using the software G* Power 3.1.9.4, considering the sample size, the OR, the probability of the event in the control group, and an α = 0.05 [25]. In addition, for MLR models, the value of the total R^2^ obtained at the end of the model was introduced for power calculation.

All statistical tests were two-sided, and differences were considered significant when *P* < 0.05 or *P* < 0.1 when *R*^2^ > 0. Statistical analyses were conducted using SPSS version 28 software (IBM Corp., Armonk, NY, USA).

## Results

### Participant characteristics

A total of 2020 participants (cases, 1012; controls, 1008) were included in the SNP and BMI analyses; given missing data for some participants, 1690 were included in the parental history analysis of T2D and 1335 in the WHR analysis. WHR was not included in some MLR models; 1690 participants were included in models where parental history was introduced. The demographic characteristics of patients with T2D and non-diabetic controls are presented in Table 1.

Among the participants, 53.9% were female and 46.1% were male. At the time of enrollment, the mean ± SD age of non-diabetic controls (59.1 ± 11.3 years) was higher than that of cases (55.5 ± 11.7 years). The mean ± SD age at T2D diagnosis was 45.8 ± 10.7 years, and the number of years with T2D varied widely (range, 0–46 years; mean ± SD, 9.5 ± 8.7 years).

### Identification of genes associated with T2D using ULR models

The allelic frequencies of 23 (Table 2) of the 69 SNPs explored (Additional file 1: Table S1) were significantly different (*P* < 0.1) between cases and controls when compared in the total sample (Additional file 1: Table S3) or stratified by age at T2D diagnosis (≤ 45 years and ≥ 46 years) (Additional file 1: Table S3), sex (male and female) (Additional file 1: Table S4), or both (Additional file 1: Table S5). In the allelic ULR analysis, only 12 of 23 SNPs showed a significant association with T2D for the total sample (Table 2). The *R*^2^ sum of these 12 SNPs only explains 5.3% of the variability of T2D etiology.

**Table 2:** Association of 23 SNPs with type 2 diabetes in the total population. (*N* = 4040 chromosomes)

Locus (SNP)	Chr	Position	RA	AA	MAF	ULR*			
						OR (95% CI)	p-Wald	*R*^2^	SI (*p*-Wald)
Group 1									
* INS-IGF2* (rs149483638)	11	2,140,300	C	T^a^	0.281	1.25 (1.1–1.4)	0.0022	0.003	0.035
* INS* (rs689)	11	2,160,994	A^a^	T	0.188	1.32 (1.1–1.5)	0.0005	0.004	0.062
* KCNQ1* (rs2237897)	11	2,837,316	C	T^a^	0.392	1.44 (1.3–1.6)	< 0.0001	0.010	0.004
* KCNQ1* (rs163168)	11	2,803,115	C	T^a^	0.436	1.39 (1.2–1.6)	< 0.0001	0.008	0.019
*SLC22A18* (rs450208)	11	2,910,751	T	G^a^	0.311	1.38 (1.2–1.6)	< 0.0001	0.007	> 0.1
Group 2									
* IGF2BP2* (rs4402960)	3	185,793,899	T^a^	G	0.163	1.28 (1.1–1.5)	0.0031	0.003	0.073
* TCF7L2* (rs7903146)	10	112,998,590	T^a^	C	0.121	1.5 (1.3–1.8)	< 0.0001	0.007	0.004
* CDKN2A* (rs10811661)	9	22,134,095	T	C^a^	0.096	1.12 (0.9–1.4)	0.29	0	> 0.1
* SLC30A8* (rs3802177)	8	117,172,786	A^a^	G	0.272	1.15 (1–1.3)	0.044	0.001	> 0.1
* HNF1A* (rs483353044)	12	120,999,288	A^a^	G	0.003	2 (0.8–5)	0.13	0.001	> 0.1
* WFS1* (rs4458523)	4	6,288,259	G	T^a^	0.233	1.07 (0.9–1.2)	0.35	0	> 0.1
* HMG20A* (rs1005752)	15	77,525,786	C^a^	A	0.419	0.94 (0.8–1.1)	0.34	0	> 0.1
Group 3									
* SLC16A11* (rs75493593)	17	7,041,768	T^a^	G	0.374	1.31 (1.2–1.5)	< 0.0001	0.006	> 0.1
* IRS1* (rs1801278)	2	226,795,828	T^a^	C	0.027	0.98 (0.7–1.4)	0.91	0	> 0.1
* FABP2* (rs1799883)	4	119,320,747	T^a^	C	0.241	0.99 (0.9–1.1)	0.89	0	> 0.1
* CAPN10* (rs7607759)	2	240,596,709	G^a^	A	0.053	1.04 (0.8–1.4)	0.76	0	> 0.1
* PPP1R3A* (rs1799999)	7	113,878,379	A^a^	C	0.291	1.09 (0.9–1.2)	0.23	0	0.006
*PTPRD* (rs10511567)	9	11,606,348	C^a^	T	0.278	1.02 (0.9–1.2)	0.76	0	0.005
Group 4									
* IL6* (rs1800795)	7	22,727,026	C^a^	G	0.079	1.01 (0.8–1.3)	0.93	0	0.033
*NOS3* (rs2070744)	7	150,992,991	C^a^	T	0.145	0.84 (0.7–1)	0.054	0.001	> 0.1
* CPED1* (rs10261386)	7	120,711,855	C^a^	T	0.347	1.04 (0.9–1.2)	0.55	0	> 0.1
* KHDRBS3* (rs6577691)	8	135,961,229	G^a^	T	0.123	0.84 (0.7–1)	0.076	0.001	> 0.1
* CACNA1H* (rs4984636)	16	1,202,441	C^a^	T	0.113	0.83 (0.7–1)	0.073	0.001	> 0.1
No. genes						12			
Sum of univariate *R*^2^						0.053			

*AA* alternative allele, *RA* risk allele, *Chr* chromosome, *CI* confidence interval, *MAF* major allele frequency, *OR* odds ratio, *R*^2^ variability of T2D explained by the SNP, *SI* sex interaction, *SNP* single nucleotide polymorphism, *ULR* univariate logistic regression

^a^Minor allele. Group 1, genes associated with insulin production located in the chromosome region 11p15.5; Group 2, genes associated with insulin production located in other chromosomes; Group 3, genes associated with peripheral insulin resistance; Group 4, genes associated with inflammation, and other functions

*The power (1 − β error probability) > 0.99 for all SNPs with *P* < 0.1

When stratified by age at T2D diagnosis (≤ 45 years versus ≥ 46 years), 8 of the 12 SNPs associated with T2D in the total sample were associated with T2D in both age groups (Additional file 1: Table S6). SNPs in *SLC30A8* (rs3802177), *NOS3* (rs2070744), and *KHDRBS3* (rs6577691) were associated only with early T2D diagnosis and SNPs in *CACNA1H* (rs4984636) were only associated with late diagnosis. Two additional SNPs, not associated with T2D in the analysis of the total sample, were also identified: *WFS1* (rs4458523) (associated with early T2D diagnosis) and *HMG20A* (rs1005752) (associated with late T2D diagnosis). No major differences were observed in the association of gene groups between early or late T2D diagnosis. However, all SNPs except *INS-IGF2* (rs149483638) had a greater association with early than late T2D diagnosis. Compared with the overall analysis, the *R*^2^ sum increased in SNPs associated with early T2D diagnosis (*R*^2^ sum = 7.37%) and decreased in those associated with late T2D diagnosis (*R*^2^ sum = 3.32%).

**Fig. 1:**
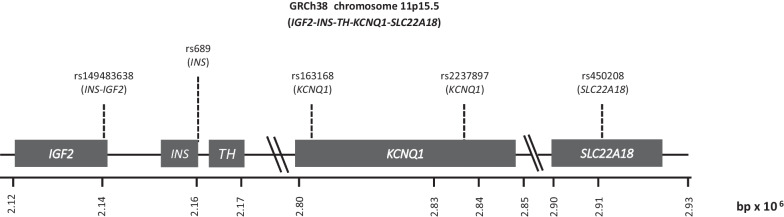
Genomic map of region 11p15.5. The figure shows the locations of genes *IGF2*, *INS*, *TH*, and *KCNQ1* and the SNPs of the region explored in this work. The positions are based on the human genome version GRCh38. bp, base pairs; SNP, single nucleotide polymorphism

There was a significant interaction (*P* < 0.1) between sex and some SNPs, both in the entire study population (*n* = 9) and when stratified by early (*n* = 9) and late (*n* = 6) T2D diagnosis (Table 2; Additional file 1: Table S6). Additional file 1: Table S4 shows allelic frequency and Additional file 1: Table S6 shows the ULR findings stratified by sex. Twelve and eight of 23 SNPs were associated with T2D in males and females, respectively, with five SNPs shared by both sexes. A large difference was observed in the association of different groups of genes between males and females. All five SNPs on the chromosome region 11p15.5 (containing genes involved with insulin production *INS*, *IGF2*, *KCNQ1,* and *SLC22A18*) were associated with T2D in males but only three in females (those located in genes *KCNQ1* and *SLC22A18*) (Fig. 1). The two SNPs located in *KCNQ1* had a stronger association with T2D in males than females (OR > 1.6 versus OR = 1.2; *P* < 0.0001 versus *P* < 0.05), while the SNP in *SLC22A18* had a slightly higher association in females versus males (OR > 1.43 versus OR = 1.32; *P* < 0.0002 versus *P* < 0.007). None of the seven SNPs in genes involved with insulin production located in other genomic regions (Group 2) were associated with T2D in females; however, three SNPs (*IGF2BP2* [rs4402960], *TCF7L2* [rs7903146], and *SLC30A8* [rs3802177]) were associated in males.

Peripheral resistance appears more important than insulin production for T2D development in females. The SNP in *SLC16A11* had a greater effect in females than males (OR > 1.37 versus OR = 1.24; *P* < 0.0004 versus *P* < 0.021) and the SNP in *PPP1R3A* was only associated with T2D in females. Overall, ORs were higher and *P* values lower in genes associated with T2D in males versus females. In females, the highest ORs were reported for SNPs in *SLC16A11* (1.37) and *SLC22A18* (1.43), while there were five SNPs with an OR > 1.5 (located in *IGF2BP2*, *INS*, *KCNQ1*, and *TCF7L2*) for males. The *R*^2^ sum was 3.5% for females and 10.7% for males. When stratified by sex and age at T2D diagnosis, some additional differences in the associations of groups of genes were seen between the sexes (Table 3 and see below in the MLR analysis).

**Table 3:** Allele association of 23 SNPs with type 2 diabetes stratified by diagnosis age and sex (*N* = 4040 chromosomes)

Locus (SNP)	ULR*							
	T2D diagnosis ≤ 45 years				T2D diagnosis ≥ 46 years			
	Females (*n* = 1662)		Males (*n* = 1404)		Females (*n* = 1596)		Males (*n* = 1384)	
	OR (95% CI)	*p*-Wald	OR (95% CI)	*p*-Wald	OR (95% CI)	*p*-Wald	OR (95% CI)	*p*-Wald
Group 1								
* INS-IGF2* (rs149483638)	1.06 (0.8–1.3)	0.61	1.38 (1.1–1.8)^a^	0.014	1.1 (0.9–1.4)	0.44	1.56 (1.2–2)^a^	0.0010
* INS* (rs689)	1.09 (0.8–1.4)	0.52	1.63 (1.2–2.1)^a^	0.0003	1.23 (1–1.6)^a^	0.11	1.43 (1.1–1.9)^a^	0.012
* KCNQ1* (rs2237897)	1.31 (1.1–1.6)	0.016	1.97 (1.5–2.5)^a^	< 0.0001	1.09 (0.9–1.4)	0.46	1.56 (1.2–2)^a^	0.0002
* CDKN1C* (rs163168)	1.36 (1.1–1.7)^a^	0.0043	1.82 (1.4–2.3)^a^	< 0.0001	1.05 (0.8–1.3)	0.64	1.45 (1.2–1.8)^a^	0.0015
* SLC22A18* (rs450208)	1.46 (1.2–1.9)^a^	0.0014	1.27 (1–1.6)	0.053	1.38 (1.1–1.8)^a^	0.0092	1.36 (1.1–1.8)	0.015
Group 2								
* IGF2BP2* (rs4402960)	1.16 (0.9–1.5)	0.26	1.65 (1.2–2.2)^a^	0.0007	1.09 (0.8–1.4)	0.54	1.37 (1–1.9)	0.042
* TCF7L2* (rs7903146)	1.22 (0.9–1.6)^a^	0.15	2.21 (1.6–3)^a^	< 0.0001	1.15 (0.9–1.5)	0.34	1.83 (1.3–2.5)^a^	0.0003
* CDKN2A* (rs10811661)	1.47 (1–2.2)^a^	0.049	0.92 (0.6–1.3)	0.65	1.07 (0.7–1.5)	0.73	1.08 (0.7–1.6)	0.72
* SLC30A8* (rs3802177)	1.31 (1–1.6)^a^	0.018	1.43 (1.1–1.8)^a^	0.0034	0.95 (0.7–1.2)^a^	0.65	0.96 (0.7–1.2)	0.77
* HNF1A* (rs483353044)	1.92 (0.4–9.5)	0.43	1.97 (0.5–7.9)	0.34	2.85 (0.6–12.8)^a^	0.17	1.53 (0.3–6.9)	0.58
* WFS1* (rs4458523)	1.08 (0.8–1.4)	0.52	1.3 (1–1.7)^a^	0.065	0.99 (0.8–1.3)	0.95	0.99 (0.8–1.3)	0.94
* HMG20A* (rs1005752)	0.97 (0.8–1.2)	0.81	1.04 (0.8–1.3)	0.73	0.87 (0.7–1.1)^a^	0.19	0.88 (0.7–1.1)^a^	0.27
Group 3								
* SLC16A11* (rs75493593)	1.51 (1.2–1.9)^a^	< 0.0001	1.34 (1.1–1.7)^a^	0.011	1.24 (1–1.5)^a^	0.048	1.14 (0.9–1.4)^a^	0.27
* IRS1* (rs1801278)	0.58 (0.3–1.2)	0.14	0.94 (0.5–1.9)	0.87	1.21 (0.7–2.2)^a^	0.53	1.35 (0.7–2.6)	0.38
* FABP2* (rs1799883)	0.89 (0.7–1.1)	0.34	1.23 (1–1.6)^a^	0.11	0.93 (0.7–1.2)	0.57	0.93 (0.7–1.2)	0.60
* CAPN10* (rs7607759)	0.76 (0.5–1.2)^a^	0.23	1.06 (0.6–1.8)	0.83	1.17 (0.8–1.8)	0.46	1.26 (0.8–2.1)	0.37
* PPP1R3A* (rs1799999)	1.36 (1.1–1.7)^a^	0.0061	0.83 (0.6–1.1)	0.14	1.25 (1–1.6)^a^	0.060	0.94 (0.7–1.2)	0.64
* PTPRD* (rs10511567)	1.28 (1–1.6)^a^	0.029	0.76 (0.6–1)^a^	0.036	1.16 (0.9–1.5)^a^	0.22	0.9 (0.7–1.1)	0.39
Group 4								
* IL6* (rs1800795)	0.8 (0.5–1.2)	0.25	1.02 (0.7–1.6)	0.94	0.82 (0.6–1.2)	0.32	1.67 (1.1–2.5)^a^	0.0093
* NOS3* (rs2070744)	0.71 (0.5–1)^a^	0.028	0.86 (0.6–1.2)^a^	0.38	0.85 (0.6–1.1)	0.28	0.99 (0.7–1.4)	0.96
* CPED1* (rs10261386)	0.95 (0.8–1.2)	0.62	1.1 (0.9–1.4)	0.42	0.94 (0.8–1.2)	0.61	1.23 (1–1.5)	0.088
* KHDRBS3* (rs6577691)	0.9 (0.7–1.2)	0.52	0.73 (0.5–1)^a^	0.083	1.02 (0.7–1.4)	0.91	0.68 (0.5–1)^a^	0.046
* CACNA1H* (rs4984636)	0.89 (0.6–1.2)	0.49	0.97 (0.7–1.4)	0.86	0.54 (0.4–0.8)^a^	0.0022	0.97 (0.7–1.4)	0.87
No. of SNPs	9		12		4		10	
Sum of univariate *R*^2^	0.056		0.14		0.02		0.079	

*CI* confidence interval, *OR* odds ratio, *R*^*2*^ variability of T2D explained by these genes, *SNP* single nucleotide polymorphism, *ULR* univariate logistic regression

^a^Indicates SNPs that remained in the multivariate model

^*^All female (*n* = 1086) and male (*n* = 930) controls were compared with both groups of cases. The power (1 − β error probability) > 0.99 for all SNPs with *P* < 0.1

The observed genotypic frequency was distributed according to the Hardy–Weinberg law in all 23 SNPs studied (Additional file 1: Table S7). Genotypic frequency is shown in Additional file 1: Table S8 (in the total sample and stratified by age at T2D diagnosis), Additional file 1: Table S9 (stratified by sex), and Additional file 1: Table S10 (stratified by age at T2D diagnosis and sex). Overall, SNPs associated with T2D in the allelic ULR analysis also showed a significant association in the ULR genotype analysis (Additional file 1: Table S11). However, in all ULR genotypic models, *R*^2^ sum values were much higher than those observed in the univariate allelic models, clearly indicating that genotypes rather than alleles should be used in MLR models.

### Degree of participation of each group of genes in the variability of T2D using MLR models

MLR analyses were performed with the four SNP groups introduced in successive blocks. Most SNPs associated with T2D by ULR, except SNP rs450208 (SLC22A18) in males and SNP rs2237897 (KCNQ1) in females, which could be in partial linkage disequilibrium with other SNPs in chromosome region 11p15.5 (Additional file 1: Table S12), remained in the MLR models (Additional file 1: Table S13).

**Fig. 2:**
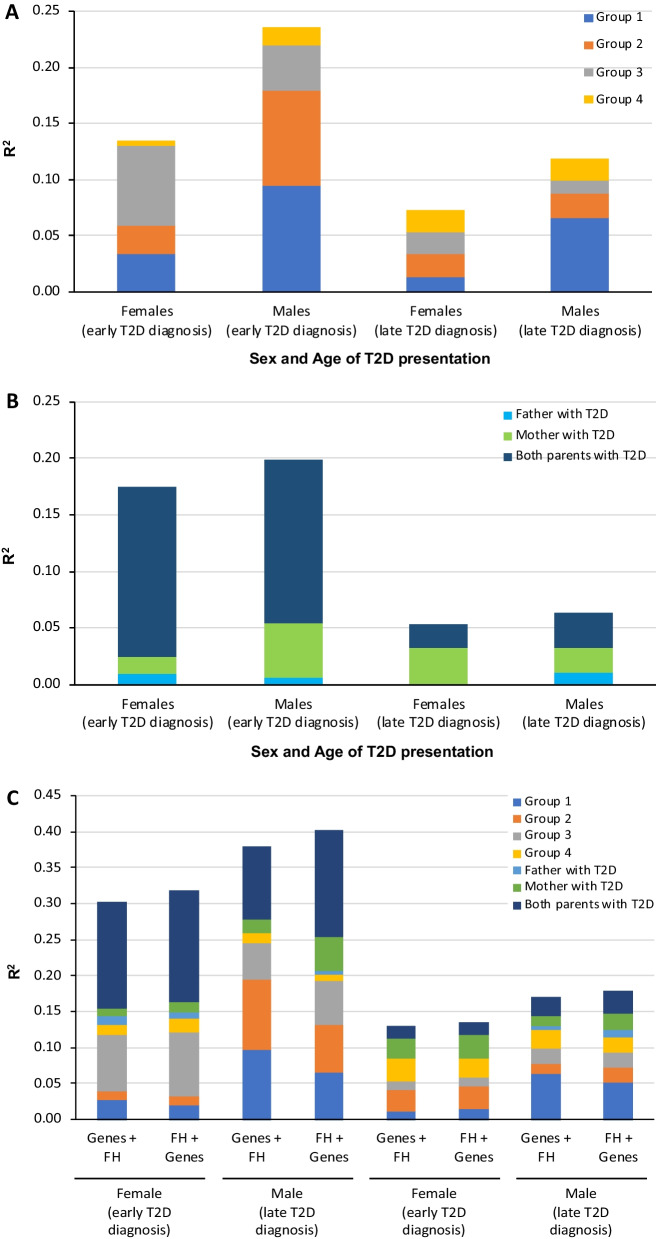
Influence (*R*^2^) of genes and parental history of diabetes on T2D development. MLR analysis stratified by age at T2D diagnosis and sex demonstrates **A** the effect of groups of genes, **B** parental history, and **C** both factors on T2D development (early diagnosis, ≤ 45 years; late diagnosis, ≥ 46 years). Group 1, genes associated with insulin production located in the chromosome region 11p15.5; Group 2, genes associated with insulin production located in other chromosomes; Group 3, genes associated with peripheral insulin resistance; Group 4, genes associated with inflammation, and other functions. The power (1 − β error probability) > 0.99 for all MLR models. *MLR* multivariate logistic regression, *SNP* single nucleotide polymorphism, *T2D* type 2 diabetes

The influence of genes on T2D was greatest in males diagnosed early (23.5%), followed by females diagnosed early (13.5%), males diagnosed late (11.9%), and females diagnosed late (7.3%). Genes on the chromosome region 11p15.5 (40.3% of *R*^2^) and others involved in insulin production (35.7% of *R*^2^) had the greatest effect in males diagnosed early (Fig. 2A), which contrasts with the major influence of genes involved in peripheral insulin resistance in females diagnosed early (52.3% of *R*^2^). Interestingly, males diagnosed late had a predominance of associated SNPs in genes of the chromosome 11p15.5 region, which contrasts with the lower *R*^2^ of the same SNPs in females diagnosed late. Although an increase in the influence of genes involved with inflammation was observed in males and females diagnosed late, it accounted for nearly one-quarter of the genes’ influence in females.

### Influence of parental history of T2D

Parental history had the strongest influence in cases diagnosed with T2D at ≤ 45 years of age (males, 19.9%; females, 17.5%) and the least influence in those diagnosed with T2D later in life (≤ 6.4%) (Fig. 2B). History of T2D in both parents had the most influence in both sexes diagnosed early. In contrast, having only a father with T2D had little or no influence on T2D development whereas having a mother with T2D influenced T2D diagnosis, especially among males diagnosed early (25%) and females diagnosed late (50%). The risk of developing T2D early was approximately 10 times higher when both parents and three times higher when one parent had a history of T2D versus neither. In contrast, a history of T2D in both parents conferred a lower risk of T2D in those diagnosed late compared with an early diagnosis (males, 3.6 times higher; females, 2.7 times higher versus neither parent with T2D) (data not shown).

In the four groups, the total value of *R*^2^ was higher when parental history was the first block and genes was the second, particularly for males diagnosed early (*R*^2^, 0.403) (Fig. 2C). In this group, gene effects decreased from 0.251 to 0.201 (Fig. 2C) as compared with genes in the first block. Contrastingly, the effect of parental history decreased when analyzing in the opposite order (*R*^2^ decreased from 0.202 to 0.119), suggesting a common effect on 5–8% of T2D variance linked to genes and parental history of T2D, and the extent of the effect of parental history is not directly due to the assessed genetic polymorphisms.

**Fig. 3:**
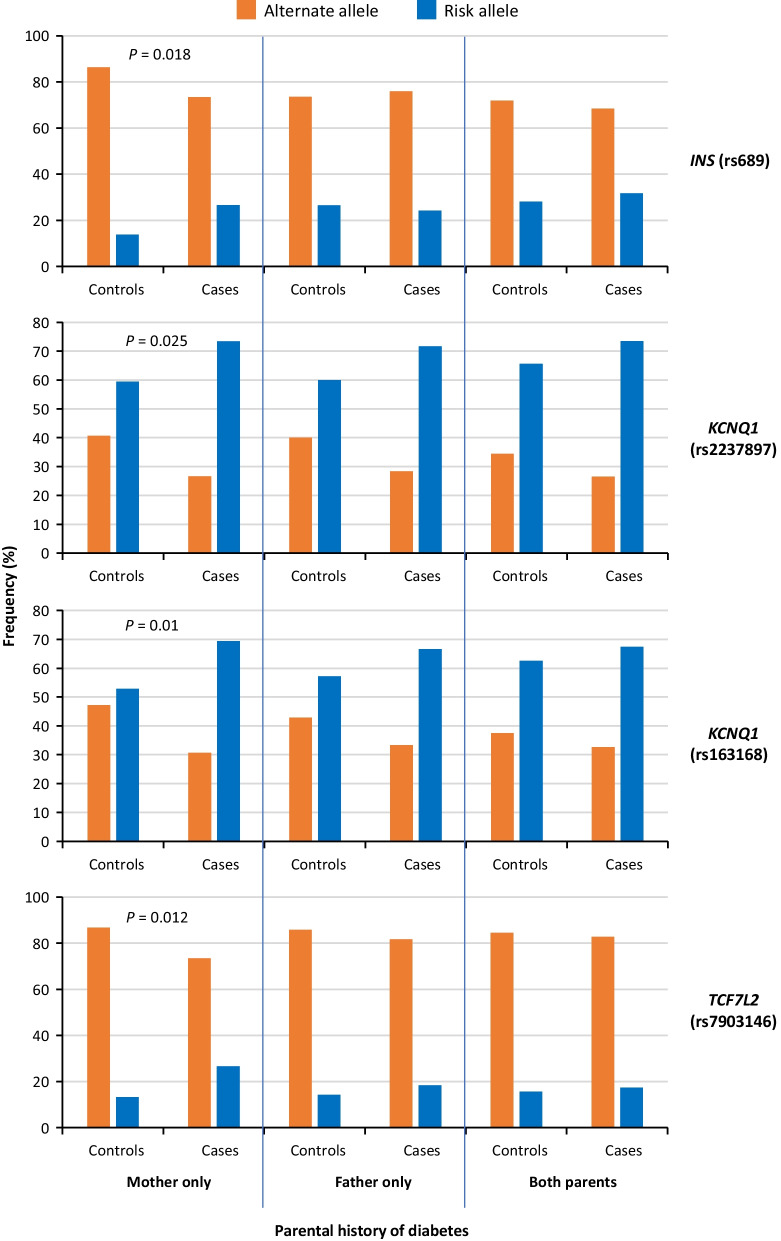
Analysis of allelic frequency stratified by type of parental history of T2D in males diagnosed early. A comparison of frequencies of risk and alternative alleles for SNPs in genes located in the chromosome region 11p15.5 (*INS* and *KCNQ1*) and *TCF7L2* between cases and controls stratified by the type of parental history of T2D (mother only, father only, both parents). *P*-value was calculated with the Chi-squared test. *SNP* single nuclear polymorphism, *T2D* type 2 diabetes

Interestingly, when the effect of genes is reduced, the reduction for the four groups of genes is not uniform. While the influence (*R*^2^) of genes related to insulin production decreased (chromosome region 11p15.5 [Group 1], 32%; other genomic regions [Group 2], 34%), the influence of genes related to peripheral insulin resistance (Group 3) increased by 25%. In the reverse model, the decreased influence of parental history was not uniform for paternal T2D history (decreased from small effect to no effect), maternal (60% decrease), or both (32% decrease) in males diagnosed early (Fig. 2C). When stratified by the type of parental inheritance, the distribution of risk alleles compared with alternative alleles for several 11p15.5 genes and *TCF7L2* only differs between cases and controls with unilateral maternal inheritance in males diagnosed early (Fig. 3).

### Influence of obesity and fat distribution

**Fig. 4:**
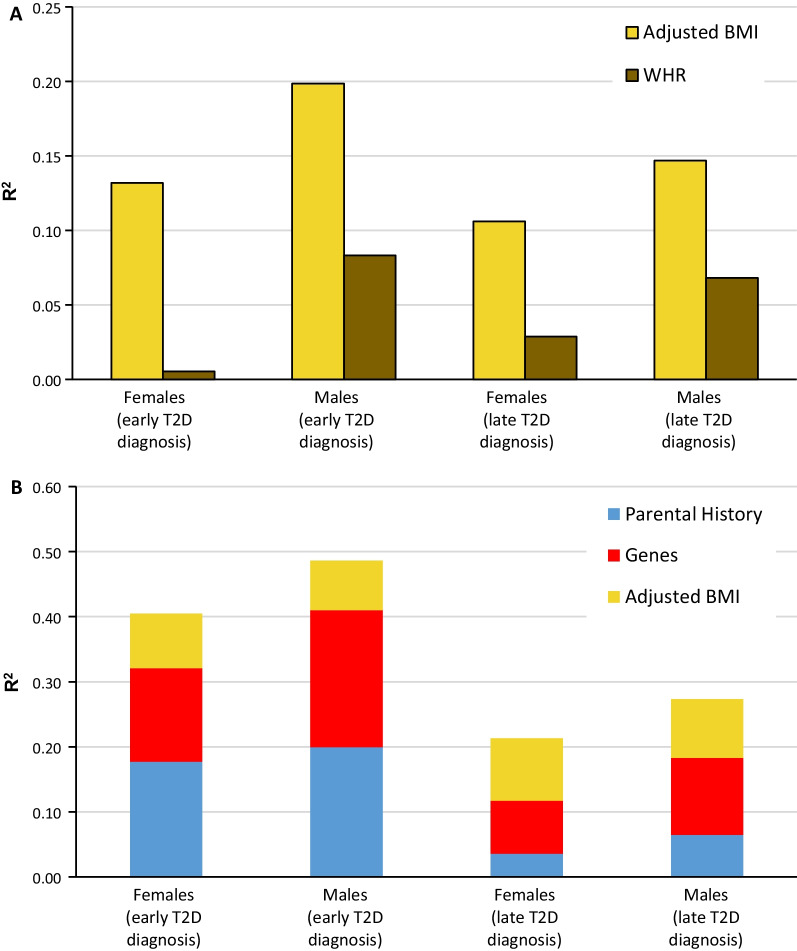
Influence (*R*^2^) of genes, parental history of T2D, BMI, and WHR on T2D development. **A** MLR analysis was conducted to determine the involvement of BMI and WHR in the four models of T2D (stratified by age of T2D diagnosis and sex). **B** MLR analysis of the participation of parental history, genes, and adjusted BMI in the four models. *BMI* body mass index, *MLR* multivariate logistic regression, *T2D* type 2 diabetes, *WHR* waist–hip ratio

**Fig. 5:**
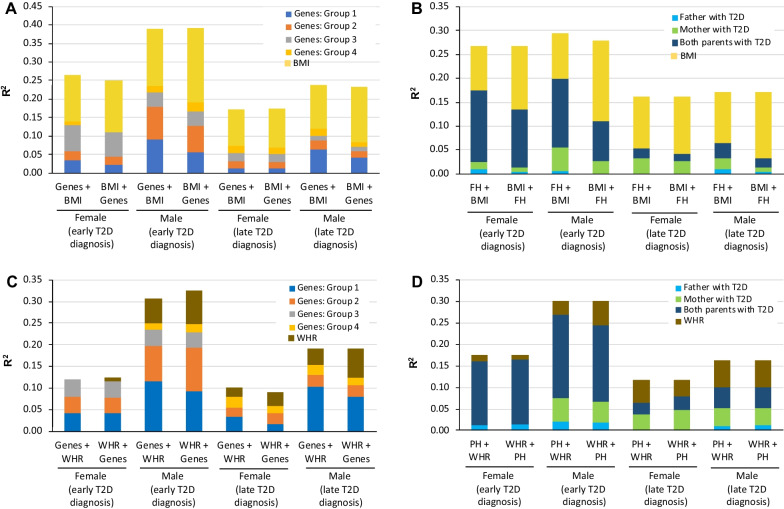
Influence (*R*^2^) of obesity and fat distribution and genes and parental history on T2D development overlap. Overlapping analysis was performed with stratification by age of T2D diagnosis and sex. Overlapping analysis of **A** BMI with genes, **B** BMI with parental history, **C** WHR with genes, and **D** WHR with parental history are shown. The overlapping effect of *R*^2^ between genes and BMI and between parental history and BMI was observed by introducing BMI as the first block and either genes or parental history as the second block and then conducting the analysis with the blocks reversed. See legend of Fig. 2 for gene groups. *BMI* body mass index, *PH* parental history, *T2D* type 2 diabetes, *WHR* weight distribution

ULR revealed that BMI was important in all four groups of T2D (males diagnosed early, *R*^2^ = 0.198; males diagnosed late, *R*^2^ = 0.147; females diagnosed early, *R*^2^ = 0.132; females diagnosed late, *R*^2^ = 0.106). WHR was most important for males irrespective of age at T2D diagnosis (Fig. 4A). BMI results were similar in MLR models when BMI was the first block (Figs. 4A, 5A, B). However, if BMI was the third block (following parental history and genes), the effect decreased substantially (males diagnosed early, 54.6%; males diagnosed late, 34.3%; women diagnosed early, 40.7%; females diagnosed late, 19.9%) (Fig. 4B).

In males diagnosed early, parental history or genes decreased the effect of BMI by 43.6% or 23.3%, respectively. With parental history as the first block, the effect was mainly absorbed by having a mother with T2D (87.2% increase) or both parents with T2D (72% increase); the effect of a father with T2D was minor (Fig. 5A, B). With genes as the first block, the effect of BMI was mainly absorbed by genes located in the chromosome region 11p15.5 (58.5% increase) (Fig. 5A). Similar increases with parental history or genes as the first block were observed in males diagnosed late and females diagnosed early (Fig. 5A, B). A similar analysis was performed using WHR, and the findings in males diagnosed early were similar to those found for BMI (Fig. 5C, D).

**Fig. 6:**
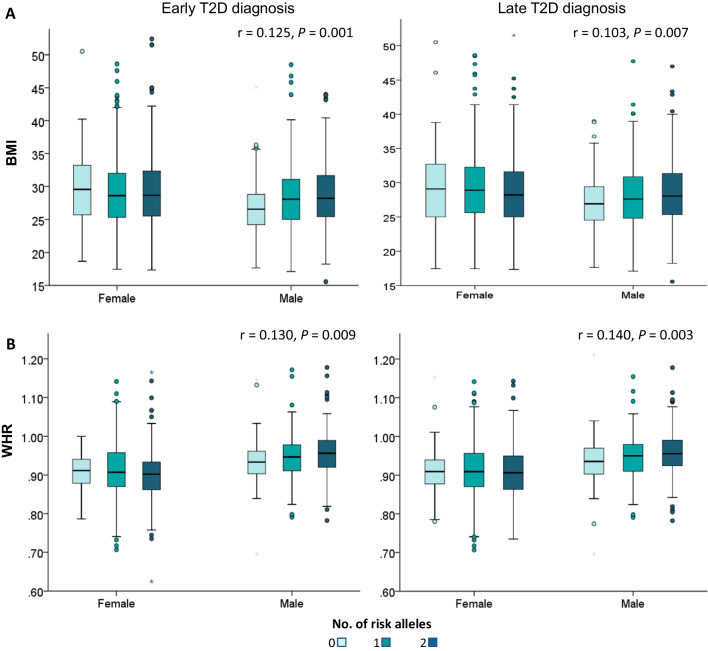
Correlation analysis between the number of risk alleles in *KCNQ1* in relation to obesity and fat distribution. **A** Correlation with BMI in males and females with a T2D diagnosis ≤ 45 years and ≥ 46 years of age. **B** Correlation with WHR in males and females with a T2D diagnosis ≤ 45 years and ≥ 46 years of age. *P*-value was calculated with the Pearson’s correlation test. *BMI* body mass index, *T2D* type 2 diabetes, *WHR* waist–hip ratio

Using the Pearson correlation, we found a positive correlation between the number of risk alleles in *KCNQ1* (chromosome 11p15.5 region) and both BMI and WHR in males diagnosed early (*r* = 0.125, *P* = 0.001 and *r* = 0.130, *P* < 0.009, respectively) and late (*r* = 0.103, *P* < 0.007 and *r* = 0.140, *P* < 0.003, respectively) (Fig. 6). A positive correlation was also observed between SNP in *INS* and BMI in males diagnosed early (*r* = 0.09, *P* = 0.019; data not shown).

When BMI was introduced as the last block, the influence of BMI was similar among the four groups (*R*^2^ range, 0.078–0.094). However, the strength of the BMI effect related to parental history and genes appeared stronger for a late diagnosis compared with an early diagnosis (Fig. 4B).

## Discussion

This study identified notable differences in the association of T2D-related genes, parental history of T2D, BMI, and WHR with T2D development between males and females of Latin American mestizo origin. The differences observed between the sexes remained regardless of whether they were diagnosed with T2D early (≤ 45 years) or late (≥ 46 years). However, in those diagnosed late, the influence of genes and family history decreased drastically, while the influence of obesity increased. The reduced effect of T2D-related genes and parental history in late-onset T2D has previously been reported in studies conducted in White populations [26, 27]. Genetic loci, parental history, and BMI appear related to each other, given that each compete for a proportion of T2D variability explained by each factor. In fact, the relationship of these three factors seems to be much more important in this population than in European populations [28].

The differences between sexes were most evident in those diagnosed early. The genetic contribution involved in insulin production was predominant in males while genes involved in peripheral resistance were more important in females; the influence of obesity, especially WHR, was much greater in males; and the influence of a mother (unilateral) with a history of T2D greatly influenced males. Because genes involved in insulin production are located in autosomes, differences in T2D association should not be related to a differential distribution of risk alleles between the sexes. Rather, this is most likely associated with other factors that differentially influence these genes in males and females. Our findings suggest that obesity, hormones, and maternal inheritance may be involved in the selection of males, but not females, who have risk alleles involved in insulin production for the early development of T2D.

The association of BMI and especially WHR was much higher in males than females in those diagnosed with T2D early (WHR, 16-fold higher). Also, in males, there was a linear correlation between BMI and WHR and the number of risk alleles in several genes associated with insulin production. Our results show that some components of parental history, and to a lesser extent, genes on the chromosome 11p15.5 region are linked to obesity, especially in males. In the literature, there is clear evidence that sex hormones play an important role in fat distribution [29]. Gluteal–femoral fat predominates in females prior to menopause, whereas abdominal fat predominates in postmenopausal females and males of all ages [30]. Abdominal fat has been associated with an increased risk of developing T2D and cardiovascular disease [31] and could contribute to the difference in the risk of developing T2D between premenopausal females compared with males in a similar age group [32].

In fact, the prevalence of T2D worldwide is higher in males than females [33, 34, 35], particularly in those aged ≤ 55 years (16.5% versus 13.5%) [17, 34, 35, 35]. In Mexico, this difference appears even greater, with the incidence of T2D reported to be 17.2% higher in males than females aged 15–49 years (524 versus 447 cases/100,000 persons) [37]. The lower prevalence in younger females potentially suggests that estrogen has a protective effect. While hormone replacement therapy results in a 35% reduction in the incidence of T2D in postmenopausal females versus placebo [38], early menopause is associated with an increased risk of T2D [39]. In fact, studies have suggested that a hormonal effect may protect or delay the impact of genes on the development of T2D in females [40–42]. Furthermore, overweight or obese males may have low concentrations of serum testosterone, which is associated with an increased risk of T2D [43, 44]. Pancreatic islets have also been shown to be more susceptible to oxidative stress in males than females [45].

The differential effect on peripheral insulin resistance between men and women appears to be associated with the SLC16A11 and PPP1R3A genes, since the effect size of the SNPs of these genes was much larger in women. SLC16A11 codes for a proton-coupled monocarboxylate transporter. Risk alleles in the SLC16A11 gene cause a decrease in gene expression and protein activity at least in hepatocytes. When the activity of the gene is abolished, the levels of acylcarnitine, diacylglycerols (DAGs) and triacylglycerols (TAGs) increase intracellularly. The levels of TAGs, which are secreted by hepatocytes in the form of VLDL (very low-density lipoprotein), are also increased at the extracellular level. These changes suggest a decrease in energy metabolism and increased lipid storage and coincide with those observed in the pathophysiology of insulin resistance and T2D [46]. Individuals with the risk alleles of SCL16A11 have decreased insulin sensitivity and an increase in the size of adipocytes in subcutaneous fat. However, the effect of the alteration in lipid metabolism appears to be much greater in women than in men, since the effect on the adipocyte size was much greater and only significant in women; in addition, only in women the distribution of abdominal fat was 3 times higher in carriers than in non-carriers of risk alleles [47].

On the other hand, disruption of the PPP1R3A gene, encoding a regulatory subunit of protein phosphatase 1 (PP1), causes a substantial decrease in glycogen synthase activity and a tenfold decrease in glycogen levels in skeletal muscle. Mice with abolished gene activity develop obesity, glucose intolerance, and insulin resistance in skeletal muscle [48]. In agreement with our study, in Maya population the rs1799999 polymorphism of the PPP1R3A gene is associated with T2D (OR = 1.625, *p* = 0.014) [49]; interestingly, the carriers of the polymorphism presented insulin resistance [49].

The influence of maternal history of T2D is more important than paternal influence, at least for early development of T2D [50]. In this study, we observed that the parental history of T2D through the mother (unilateral) may play an important role in differences in the association of risk alleles between the sexes in early-diagnosed T2D. Risk alleles in *KCNQ1* confer a risk for T2D only when inherited by the mother [51] and influence methylation levels of regulatory sequences in fetal human pancreas, suggesting that some diabetes risk effects may be mediated in early development [52]. Interestingly, the effect of maternal inheritance on genes from the 11p15.5 region was only observed in males with an early T2D diagnosis, which is an association that has not been previously reported.

There were also important differences in the T2D models of the sexes with a late T2D diagnosis. The effect size of T2D-related genes was larger in males than females, primarily at the expense of the genes involved with insulin production from the chromosome 11p15.5 region. It is also noteworthy that in both groups with a late diagnosis, the proportion of genes involved with inflammation and other processes, relative to the total variance, was higher than in those with an early diagnosis. This could be attributable to increased inflammatory processes related to aging [53].

This study had some limitations. The number of participants with missing data for WHR was notable. However, the important difference in the median WHR and the degree of association with T2D between the sexes, and the significant correlation between WHR and the number of risk alleles of the *KCNQ1* gene were notable. Furthermore, in all MLR models where WHR was introduced as a variable the statistical power [1 − β error probability] was > 0.99. However, because this study included only Mexican participants, the study findings are not necessarily generalizable to other populations.

### Perspectives and significance

Besides the differential effect of hormones, adiposity, and maternal inheritance on the development of T2D before the age of 46, between males and females, the findings of this work add the differential influence of genes. To confirm these findings, we are analyzing 92 genes associated with T2D (personal communication, Dr. Jason Torres) in 140,000 Mexicans from the Mexico City Prospective Study (MCPS) [54].

## Conclusions

The results of the present study demonstrate the greater influence of T2D-related genes, maternal T2D history, and fat distribution on T2D development in males compared with females. Insulin production-related genes were more influential in males while peripheral insulin resistance and inflammation related genes were more influential in females. The differences between males and females were seen mainly when the T2D was diagnosed before the age of 46. These results could explain the higher prevalence of T2D in men than in women, particularly in those aged 50 or younger.

## Supplementary Information

**Additional file 1. **Supplementary Tables.

## Data Availability

All data generated or analyzed during this study are included in this published article and its Additional files.
